# Spinal metastasis in thyroid cancer

**DOI:** 10.1186/1758-3284-4-39

**Published:** 2012-06-25

**Authors:** Sami Ramadan, Mohamed A Ugas, Richard J Berwick, Manisha Notay, Hyongyu Cho, Waseem Jerjes, Peter V Giannoudis

**Affiliations:** 1 Department of Medicine, University College London Medical School, London, UK; 2 Barts and The London School of Medicine and Dentistry, University of London, Queen Mary, London, UK; 3 Department of Surgery, Al-Yarmouk University College, Baghdad, Iraq; 4 Department of Surgery, UCL Medical School, London, UK; 5 Leeds Institute of Molecular Medicine, University of Leeds, London, UK; 6 Academic Department of Trauma and Orthopaedic Surgery, Leeds Teaching Hospitals NHS Trust, Leeds, UK

## Abstract

**Background:**

Thyroid carcinoma generally responds well to treatment and spinal metastasis is an uncommon feature. Many studies have looked at the management of spinal metastasis and proposed treatments, plans and algorithms. These range from well-established methods to potentially novel alternatives including bisphosphonates and vascular endothelial growth factor (VEGF) therapy, amongst others.

The purposes of this systematic review of the literature are twofold. Firstly we sought to analyse the proposed management options in the literature. Then, secondly, we endeavoured to make recommendations that might improve the prognosis of patients with spinal metastasis from thyroid carcinomas.

**Methods:**

We conducted an extensive electronic literature review regarding the management of spinal metastasis of thyroid cancer.

**Results:**

We found that there is a tangible lack of studies specifically analysing the management of spinal metastasis in thyroid cancer. Our results show that there are palliative and curative options in the management of spinal metastasis, in the forms of radioiodine ablation, surgery, selective embolisation, bisphosphonates and more recently the VEGF receptor targets.

**Conclusions:**

The management of spinal metastasis from thyroid cancer should be multi-disciplinary. There is an absence; it seems, of a definitive protocol for treatment. Research shows increased survival with ^131^I avidity and complete bone metastasis resection. Early detection and treatment therefore are crucial. Studies suggest in those patients below the age of 45 years that treatment should be aggressive, and aim for cure. In those patients in whom curative treatment is not an option, palliative treatments are available.

## Introduction

Thyroid cancer is uncommon, accounting for roughly 1% of all new malignant disease, about 0·5% of cancers in men and 1·5% in women [1-4]. It affects approximately 1900 people in the UK each year, but its incidence has been increasing for decades [5-8]. Much of the rise in incidence can be explained by improved diagnostic methods. However, this cannot entirely explain the upward trend [8-10].

Differentiated thyroid cancer (DTC) account for the vast majority (90%) of all thyroid cancers and includes papillary (70–75%) and follicular (15–20%) cancers. Hürthle-cell cancers (2%) and insular carcinomas also arise from follicular cells but are probably within the spectrum from differentiated to undifferentiated. Undifferentiated carcinomas, which are anaplastic malignancies, account for <5% of thyroid cancers. They derive from follicular cells. Medullary carcinoma, in contrast, constitutes 5–10% of thyroid cancers [5,11,12].

The significance of the histological distinction relates to the management and prognosis of the different types of carcinomas. Differentiated carcinomas have a better prognosis than undifferentiated, Hürthle and insular cancers [8,11]. Medullary thyroid carcinomas arise from the C-cells and are, therefore, aetiologically and clinically, different to follicular cancers [8,11]. There are also other rare types of thyroid malignancies including lymphomas and sarcomas.

In general, thyroid cancer is more prevalent in the middle-aged or old and in females [4]. Papillary malignancy typically occurs in young females and sometimes in children, whilst Follicular malignancy predominantly affects middle-aged females. Anaplastic thyroid cancer is more common in the elderly [7].

Papillary carcinoma is characterised by indolence and localised spread. It is known, however, to metastasize to both lung and bone. The prognosis of papillary thyroid cancer is good, especially in the young and if diagnosed early. Follicular malignancy preferentially metastasises to lung and bone. The prognosis is good if diagnosed early. Anaplastic thyroid carcinomas are very aggressive and locally invasive. They respond poorly to treatment and prognosis is poor. Medullary cancer sometimes has a familial origin associated with the RET gene and can also be part of Multiple Endocrine Neoplasia type 2 syndrome (MEN-2 syndrome). It is too, characterised by indolence and localised spread, as well as metastasis to different sites. It has a poor prognosis but can be treated if found before distant spread [3,5,12,13].

In 90% of cases, thyroid carcinoma presents simply as thyroid nodules. Rarely, the first manifestation is cervical lymphadenopathy (5%), or distant metastasis in the lungs, bone, liver or brain. Bony metastasis occurs in approximately 2–13% of people with thyroid malignancy; the proportion is overwhelmingly follicular, and many of those develop in the spine [3,13,14]. The latter are particularly debilitating as they often cause pain, fractures, spinal cord compressions and affect mobility and quality of life (QoL) [15]. The remission rate in bony metastasis is 7–20% [16].

The survival rate in thyroid carcinoma is generally good, apart for the anaplastic type. The 10-year survival rate in DTC is 80–95%. However, this figure drops to about 40% when distant metastasis is present [16].

The pathogenesis of thyroid carcinomas is not fully understood. Aetiology is known only in a few rare cases of familial papillary and medullary carcinomas, and in some patients who have been exposed to irradiation or radioactivity [5].

There is a pathway to diagnose thyroid cancer including: physical exam to feel for nodules, blood tests (particularly: TSH, thyroglobulin and calcitonin levels), ultrasound, thyroid scan with a radioactive marker. Also, biopsy with fine needle aspiration (FNA) is helpful; however, surgery is the gold standard and the only conclusive diagnostic method [4,10,17].

### Management of thyroid cancer

It is widely agreed in the literature that surgical resection should be the initial step in treating most thyroid cancers [4,17-19]. Following a biopsy to determine the histology of the tumour, a decision is made whether to perform a lobectomy or total thyroidectomy [4,19]. Patients who have had total or near-total thyroidectomy are required to have their serum thyroglobulin checked no sooner than six weeks after the procedure in order to check for recurrence.

The surgery is often followed by adjuvant radioiodine therapy (^131^I ablation). Following the resection of the thyroid gland, thyroid hormone replacement therapy is given in order to rectify the resulting hypothyroidism. It is also thought that in high doses the replacement therapy helps prevent recurrence of the cancer [19].

Chemotherapy and external radiation are also used in the treatment albeit more rarely than the above mentioned treatments. External radiation is used infrequently as palliative treatment in cases of unresectable or secondary disease [10,11,17,19].

The use of chemotherapy is more controversial. It is generally ineffective in most types of thyroid cancers, but it is indicated for the anaplastic type, lymphoma or metastatic tumour. It has also been used as palliation in end-stage disease unresponsive to irradiation and also for progressive, symptomatic disseminated cancer [20]. The agents used are doxorubicin and cisplatinum [11,17,19].

In most cases the treatment of thyroid carcinomas results in long-term remission. Survival is excellent in younger patients with recurrence more likely at extremes of age [21,22]. However, there are still patients with poor outcomes. They are usually the patients with metastasis and/or advanced disease at diagnosis [11,18]. For this reason, new treatment modalities are being explored.

### Metastasis from thyroid cancer

DTC metastasises preferentially to bone (25%) second only to the lung (49%) whilst synchronous spread to lung and bone accounts for a further 15% of distant metastasis of DTC. The remaining occur in other soft tissues [3,23,24].

Bone metastasis occurs in 2–13% of DTC cases. They are more prevalent in follicular cancer (7–28%) than in papillary cancer (1.4-7%) [3]. The great majority of bone metastasis occur in regions where blood flow is high, such as the axial skeleton red marrow in vertebrae, ribs and hips [3]. The spinal lesions are mostly osteolytic, with new bone forming in response to the bone destruction [3].

The diagnostic modalities used in spinal metastasis are different to those used in the determination of thyroid cancer. CT and MRI scans are used to provide high resolution imaging of the spine. MRI is better at differentiating soft tissue structures and, therefore, it is slightly more sensitive at detecting bone marrow metastasis^26^. Bone scintigraphy and other nuclear studies, such as FDG-PET and SPECT, are also used for localisation of lesions and have high sensitivity and specificity [25].

When bony metastasis is present, the 10-year survival rate is estimated between 13% and 21%, which is roughly equivalent to a 50% reduction compared to non-metastasised cancer^3^. It is often the case that the lesion(s) do not respond to curative treatment and palliative treatment at diagnosis remains the only option; the aim being to improve the quality of life [3].

### Spinal metastasis

Bone, after the lungs and liver is the third commonest site of metastasis [26]. Spinal metastasis is by far the most common type of spine tumours, outnumbering primary spinal neoplasm by more than twenty-to-one [25], and, are overwhelmingly breast, lung, prostate or renal primaries in origin [25-27]. This owes much to their respective incidence as well as their special predilection for the vertebral column [25].

Prevalence of spinal metastasis (SM) is highest among individuals between the 4^th^ and 7^th^ decade of life [25,27,28]. Males are more likely to be afflicted; this is thought to be reflective of the higher prevalence of lung cancer in this group [12] and the higher prevalence of prostate cancer relative to breast cancer in females [25].

At the time of initial diagnosis, 1%-3% of patients with thyroid cancer have distant metastasis whereas another 7%-23% will develop distant metastasis during the course of their disease (2,5-8,10-12). Bone metastasis is diagnosed clinically in 2%-13% of patients with differentiated thyroid cancer [29].

Well-differentiated thyroid cancers (DTC) account for the vast majority (85–98%) of thyroid malignancies. Bone metastasis incidence in differentiated thyroid cancers (DTC) is 2–13% [30,31]. Papillary thyroid cancers (PTC) accounts for 77% of DTC and has a low incidence of SM of 1–7% [31] whilst follicular thyroid cancer (FTC) which accounts for 15% of all DTC has an incidence of bone metastasis of 7–20% [31]. Hürthle cell carcinomas, which accounts account for 2% of thyroid malignancies, have the highest propensity (12%) to metastasize to spine.

Spinal metastasis typically affect the thoracic (60–80%), lumbar (15–30%) and cervical spine (<10%) [32-54] with the preferred route of metastasis to the spine being via the arterial or venous -Batson’s venous plexus - vessels resulting in multifocal lesions. Direct infiltration from paraspinous disease or, less commonly, through the cerebrospinal fluid [25,32] are also potential routes of metastasis. The vertebral body (85%) [54] is the commonest site for initial involvement; the posterior aspect of which is preferentially involved (66%) [25]. The paravertebral spaces (10–15%) and the epidural space (<5%) are also initial sites of metastatic involvement [25,32,54].

Vertebral metastasis are asymptomatic and may be incidental findings following routine bone scans in patients presenting with systemic disease [32,33]. Classical clinical symptoms develop with the progression of spinal metastatic disease and are consequences of metastatic infiltration and/or compression of paravertebral, osseous and neural tissue [33].

Spinal canal to spinal cord ratio is smallest in the thoracic spine hence spinal cord compression is more common in the thoracic spine [33]. The most frequent cause of spinal cord compression and nerve root compression is the expulsion of metastatic tissue and/or detritus of bone into the spinal canal or neural foramina following metastatic infiltration and ensuing partial collapse of the vertebral body. On infrequent occasion, metastatic tissue may break into the spinal canal and cause spinal cord compression without assaulting the vertebral body’s structural integrity.

The chief presenting symptom of spinal metastasis is pain (83–95%) [25,32]. Patients often present with localised, gradual onset pain caused by periosteal stretching and inflammation [32]. It is invariably progressive and unremitting in nature, often worse at night. It may improve with activity and anti-inflammatory medications. The affected area is typically tender on examination [33].

Radicular pain, described as shooting pain, is a common compliant in SM. It is caused by impingement or irritation of nerve roots in the intervertebral foramina, either by direct tumour compression or via tumour induced pathological fractures. Neuropathic pain characterised by an intense, burning sensation is more common in intradural metastasis [32].

Metastatic invasion of the spinal canal occurs predominantly in an anterior direction. Spreading via the posterior aspect of the vertebral body [33], deficiencies in motor functions comprise the second most common presenting symptom (35–75%^26^) in patients with vertebral column metastasis [32,33]. Limb weakness is a common symptom, which is seen also on physical examination [25].

Posterior displacement of the spinal cord and impingement against the lamina may result in sensory dysfunction [32,33]. It is a feature of advanced spinal metastatic disease and can be accompanied by profound motor dysfunctions such as anal and urethral sphincter dysfunction and sexual malfunction [25,32].

These “red flag” signs and symptoms should prompt SM diagnosis in thyroid cancer patients. In such patients, a full diagnostic workup should be instigated to ascertain the level(s) of vertebral column involvement, spinal instability and the degree of neurological impairment. These parameters influence the decision to operate or not [25,32,33].

Plain X-ray is used to identify metastatic lesion(s), tumour mass(s) and evaluation of spinal stability [27,32]. Spinal instability is indicated by progressive deformity, significant angulation or translocation and/or >50% involvement of the vertebral body. Cervical disk space narrowing is an important maker of vertebral collapse. Metastatic lesions are indicated by vertebral body involvement, erosion of the spinous process and the inability to fully visualize pedicles [27]. X-rays are insensitive in early spinal metastatic diagnosis, however, because a 30–50% demineralisation of bone is required before lytic lesions become apparent on radiographic film [25,27,32].

Magnetic resonance imaging (MRI) is the gold-standard imaging modality in SM diagnosis. It renders exquisitely detailed multiplanar imaging, allowing the visualisation of metastatic infiltration and/or compression of paravertebral, osseous and neural tissue [25,27,32]. T1- and T2-weighted imaging as well as contrast-enhanced and fat-suppressed studies in all three planes aid diagnosis^33^.

Computed tomography (CT) imaging is an excellent modality in assessing the osseous spine. It has a high degree of accuracy (90% sensitivity, 100% specificity) [28] in identifying metastatic lesions, vertebral destruction and spinal stability. CT angiography is ideal in identifying spinal metastasis from highly vascular primary malignancies such as thyroid cancers [32].

Bone scintigraphy is used to screen for bone metastasis. Despite its documented 62–89% [25] sensitivity, it should be noted that bone scintigraphy measures abnormalities in bone metabolism and does not, therefore, possess a high specificity in identifying SMs.

MRI and/or CT should be used to authenticate suspected SM. Single-photon emission computed tomography (SPECT) and fluorodeoxyglucose positron emission tomography (FDG-PET) [25] are both superior to bone scintigraphy and are used in surveillance of patients suspected of SM. Finally biopsy under CT fluoroscopic guidance is crucial in staging SM and formulating surgical treatment plan.

The purposes of this systematic review of the literature are twofold. Firstly we sought to analyse the proposed management options in the literature. Then, secondly, we endeavoured to make recommendations that might improve the prognosis of patients with spinal metastasis from thyroid carcinomas.

## Materials and methods

A thorough electronic literature search was conducted using online journal databases. These included PubMed, Google Scholar, Web of Knowledge, Science Direct and MEDLINE. Our search criteria terms were broad including, “spinal metastasis in thyroid cancer”, “management of spinal metastasis”, “thyroid metas* spin*”. This yielded 20 studies. To supplement our research, we reviewed the references of the literature initially found, in order to avoid neglecting relevant articles that were missed by our search criteria. We excluded from our initial pool of recruited studies those that were review papers and those articles dated pre 1990 to ensure a body of contemporary literature.

Articles were deemed pertinent if they described metastatic thyroid cancer of a follicular, papillary or Hürthle nature. We sought, particularly, studies that included a population of patients with bone metastasis and spinal involvement. We did not include small case reports or studies that investigated spinal metastasis, without mention of thyroid cancer because the nature of thyroid cancer treatment is specific. In order to attain reliably representative results, we eliminated case reviews. The chosen studies were selected by two independent reviewers using the above protocol.

## Results

**Figure 1:**
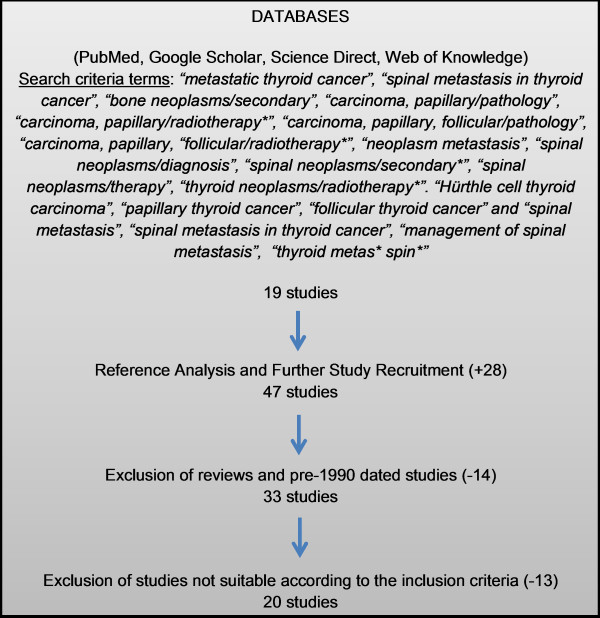
Flow chart illustrating search protocol.

Our search protocol refined our pool of articles to 20 [13-16,29,30,34-47], as seen in Figure 1. In this systematic review we focused on therapeutic management of spinal metastasis in thyroid cancer with reference to survival time, pain management and prognostic indicators. As a part of this review, the various parameters were evaluated including the study design, sample size, histological type of cancer, mean age, outcome and mortality. The characteristics of the studies can be seen in Table 1. 

**Table 1:** Summary of the study characteristics

**Study, year**	**Study design**	**End point**
Bernier *et al.*, 2001^35^	Retrospective cohort analysis	Survival and impact of treatment on survival.
Cabanillas *et al.*, 2010^36^	Prospective intervention	Determine response and progression free survival due to treatment with sorafenib/sunitinib
De Vries *et al.*, 2008^37^	Retrospective cohort analysis	Determine the effect of selective embolisation on serum thyroglobulin and symptoms
Demura *et al.*, 2011^14^	Retrospective cohort analysis	Determine the effect of total en bloc spondylectomy on survival and local recurrence
Durante *et al.*, 2006^31^	Retrospective cohort analysis	Determine the response and effect on survival of ^131^I therapy
Eustatia-Rutten *et al.*, 2003^17^	Retrospective cohort analysis	Determine the effect of palliative embolisation on clinical symptoms.
Hindie í *et al.*, 2007^15^	Observational study	To investigate the impact of early ^131^I-based detection on outcome.
Kloos *et al.*, 2009^38^	Phase II Trial	Objective response rate of VEGFR inhibitors
Orita *et al.*, 2011^16^	Retrospective cohort analysis	Determine the efficacy of zolendronic acid on skeletal-related events and bone metastasis from thyroid cancer
Orita *et al.*, 2010^39^	Retrospective cohort analysis	Determine prognostic factors and analyse treatment methods
Petrich *et al.*, 2000^40^	Retrospective cohort analysis	Determine the therapeutic outcome and side effects of radioiodine in bone metastasis from thyroid cancer
Pittas *et al.*, 2000^30^	Retrospective cohort analysis	Determine prognostic indicators and impact of treatment on survival
Quan *et al.*, 2012^41^	Retrospective cohort analysis	Determine survival of patients with vertebral metastasis from thyroid cancer
Qiu *et al.*, 2011^42^	Retrospective cohort analysis	Determine the efficacy of ^131^I therapy and survival
Schlumberger *et al.*, 1996^43^	Retrospective cohort analysis	Determine the response of bone and lung metastases to ^131^I
Stojadinovic *et al.*, 2002^44^	Retrospective cohort analysis	Determine distant disease free survival and post metastatic disease specific survival after surgery for distantly metastatic thyroid carcinoma
Van Tol *et al.*, 2000^45^	Retrospective cohort analysis	Determine the effect of embolisation in combination with radioiodine in terms of pain, serum T and neurological symptoms
Vitale *et al.*, 2001^46^	Retrospective cohort analysis	Determine the impact of pamidronate on bone pain, performance status and evaluate the degree of tumour response.
Wells *et al.*, 2012^47^	Phase III RCT	Determine progression free survival of patients on vandetanib for metastatic thyroid cancer
Zettinig *et al.*, 2002^48^	Retrospective cohort analysis	Determine the role of surgery on survival of metastatic thyroid cancer

Our search protocol retrieved 20 studies published from 1996 to 2012 (Table 1). Studies investigated the use of conventional and novel intervention for distant and spinal metastasis, including radioactive iodine, surgery, selective embolisation therapy (SET), small molecule inhibitors and bisphosphonates. Reported outcomes included survival rates, morbidity and prognostic factors associated with each intervention. There were 16 retrospective cohort studies [13,15,16,29,30,34,36,38-45,47], 1 observational study [14], 1 prospective interventional study [35], 1 Phase II trial [37] and 1 Phase III clinical trial [17].

Reported cases of spinal metastasis and therapeutic modalities used can be seen in Table 2. Summary results pertaining to the use of conventional and novel therapeutic modalities in treating patients with distant metastasis from thyroid carcinoma can be seen in Tables 3 and 4, respectively. The population size in each study varied considerably between studies, from Durante *et al.*[30] (n = 444) to Van Tol *et al.*[44] (n = 11). 

**Table 2:** Clinical characteristics at diagnosis of patients with spinal metastasis from thyroid carcinoma and the therapeutic strategies deployed

**Author, Year**	**Patient No.**	**Age (yrs), sex at time of symptomatic spine disease**	**Thyroid cancer histology**	**TNM stage**	**Intervention for primary site**	**Location of spinal metastasis**	**Signs + symptoms of spinal metastasis/indication for surgery**	**Intervention for spinal metastasis**	**Outcome at last follow-up (months)**
Quan *et al.*, 2012	1	76, F	FWD	T2 N0 M1	Total thyroidectomy followed by 131-I (100 mCi) therapy every 3–9 months for 2 years then once a year till disappearance of metastatic uptake	L3	Intractable pain resistant to non-operative measures and pain and/or paralysis due to bony instability or spinal cord compression by metastatic tumour	L1-4 decompression and stabilization	AWD (60)
	2	76, F	FWD	T4 N0 M1		Lumbar		L4-5 decompression and stabilization	AWD (56)
	3	60, F	PWD	T1 N0 M1		L4		L3-5 decompression and stabilization	AWD (20)
	4	67, F	FWD	T4 N1 M1		Thoracic		T3 anterior corpectomy and reconstruction with cement and anterior plate	CR (96)
	5	45, M	Papillary PDTC, refractory to I-131	T3 N0 M0		Thoracic	Intractable pain focal mechanical pain resistant to non-operative measures and due to osteolytic metastases in the absence of significant spinal cord compression or spinal instability.	T8 vertebroplasty, followed by T8-T9 decompression and then T6, T7, and L3 vertebroplasties	DOD (6)
	6	70, F	PWD	T3 N0 M1		Thoracic		T9 and L1 vertebroplasty	AWD (48)
	7	61, F	PWD	T3 N1 M0		Cervical		C3, non-operative	AWD (17)
	8	68, F	MTC	T4 N0 M1	Total thyroidectomy	Thoracic		T12 vertebroplasty	DOD (18)
Demura et al., 2011	1	55, F	Follicular	-	7/10 patients had undergone previous total thyroidectomy. Subtotal thyroidectomy was performed in 2 patients. Emergency spinal surgery (TES) was performed due to acute paraparesis in 1 case, followed by total thyroidectomy. Preoperatively, 131-I therapy had been administered in 4 cases (patient 2, 3, 5, and 9); all 4 of these patients showed a poor response to the treatment.	L2	Preserved motor function, abnormal gait and muscle weakness	SET, TES & intraoperative chemotherapy	DOD (77)
	2	56, F	Follicular	-		T7	Preserved motor function and normal gait	SET, TES & intraoperative chemotherapy	NED (125)
	3	58, F	Papillary	-		T2	Preserved motor, non-functional	SET, TES & intraoperative chemotherapy	DOD (27)
	4	56, F	Follicular	-		L2	Normal motor function	SET, TES & intraoperative chemotherapy	AWD (86)
	5	59, M	Papillary	-		T10	Normal motor function	SET, TES & intraoperative chemotherapy	DOD (62)
	6	63, F	Follicular	-		T10-11	Preserved motor, non-functional	SET, TES & intraoperative chemotherapy	AWD (56)
	7	39, F	Medullary	-		T2, T5-7	Normal	SET, TES & intraoperative chemotherapy	AWD (52)
	8	77, F	Follicular	-		T7, T10	Preserved motor function, abnormal gait and muscle weakness and bladder paralysis	SET, TES & intraoperative chemotherapy	AWD (45)
	9	57, F	Follicular	-		T11	Normal motor function	SET, TES & intraoperative chemotherapy	AWD (36)
	10	52, F	Papillary	-		L2-4	Normal motor function	SET, TES & intraoperative chemotherapy	NED (36)
De Vries *et al.*, 2008	1	60, F	FWD	T1 N0 M1	Total thyroidectomy and I-131 therapy	L4	Pain and neurological symptoms	SET, external beam radiotherapy & corporectomy L4	Symptoms decreased during 17 sessions (mean duration of 8.1 months), were unchanged during 8 sessions, and increased during 6 sessions (p < 0.01).
	2	62, F	FWD	T2 N0 M1		T5-7	Pain and neurological symptoms	SET & external beam radiotherapy	
	3	65, F	FWD	T2 N0 M1		L3- 4	Pain and neurological symptoms	SET	
	4	40, F	FWD	T4 N0 M1		L5	Pain and neurological symptoms	SET & external beam radiotherapy	
	5	77, F	FWD	T2 N0 M1		L2	Pain and neurological symptoms	SET & external beam radiotherapy	
	6	54, F	FWD	T2 N0 M0		T10-11	Pain and neurological symptoms	SET & external beam radiotherapy	
	7	35, M	FWD	Tx N0 M0		L2-4	Pain and neurological symptoms	SET & external beam radiotherapy	
	8	52, F	FWD	Tx N0 M1		L3	Pain and neurological symptoms	SET & corporectomy L3	
	9	45, F	FWD	T3 N0 M1		C4	Pain and neurological symptoms	SET & corporectomy C4	
	10	83, M	FWD	Tx N0 M1		C5-6	Pain and neurological symptoms	SET & surgical C5–C6	
Hindie í *et al.*, 2007	1	27, F	FWD (Hurthle areas)	T2 NX	Total thyroidectomy and 131-I ablation therapy (100 mCi)	T2	Back pain	Spinal surgery + radiotherapy	PR (43)
	2	34, F	PWD	T3 NX		L4	Asymptomatic, metastases revealed via 131-I ablation therapy	-	CR (8)
	3	48, M	PWD	T1 NX		T6, L2	Asymptomatic, metastases revealed via 131-I ablation therapy	-	CR (37)
	4	53, M	Follicular, PDTC	T4a N1b		T6	Back pain	Spinal surgery + radiotherapy	DOD (76)
	5	55, M	PWD (Hurthle areas)	T1 Nx		T11, L3	Asymptomatic, metastases revealed via 131-I ablation therapy	-	CR (23)
	6	63, F	FWD	T2 NX		T10-11	Back pain	Spine radiotherapy	DOD (32)
	7	75, F	FWD	T3 N0		T12	Back pain	Spinal surgery + radiotherapy	PD (62)
	8	65, F	PWD	T4b N1b		C6	Asymptomatic, metastases revealed via 131-I ablation therapy	Spine radiotherapy	DOD (34)
	9	73, M	Hürthle	T3 N1b		Cervical	Bone pain	Spine radiotherapy	DOD (5)
Eustatia-Rutten *et al.*, 2003	1	69, M	FTC, DTC	T4 N1 M1	Total thyroidectomy and 131-I ablation therapy (76–162 mCi) ± second 131-I therapy, irradiation and/or laminectomy	L1-4	Radicular pain/SSC, hyperaesthesia	SET and 131-I ablation therapy (76–162 mCi), laminectomy and irradiation	DOD (54)
	2	51, M	FTC	Tx Nx M0		T1	Radicular pain	SET	DOD (124)
	3	72, F	PTC	T4 N0 M1		T1	Paresis, incontinence	SET then laminectomy & irradiation	DOD (1.5)
	4	71, F	PTC	T4 N0 M1		L3	Radicular pain/SSC,	SET then irradiation	PD (139)
	5	62, M	FTC	T4 N0 M0		T10, T12	Radicular pain	SET	DOD (63)
	6	60, M	FTC	T2 N0 M1		C7	SSC, paraplegia, radicular pain	SET then 131-I therapy (76–162 mCi) and irradiation	DOD (86)
	7	53, F	FTC	T2 N0 M0		L1	Radicular pain, hyperaesthesia, paraesthesia	SET	PD (108)
	8	45, M	FTC	T3 N0 M0		T1-3, T5-7	Radicular pain, paraparesis, ataxia, paraplegia	SET then 131-I ablation therapy (76–162 mCi), laminectomy and irradiation	PD (152)
	9	72, M	PTC	Tx N1 M1		T9-10	Radicular pain,	SET	DOD (12)
	10	62, M	PTC	T3 N0 M0		T9-10	paraparesis, incontinence, hyperaesthesia, paraplegia, radicular pain	SET	DOD (102)
van Tol *et al.*, 2000	1	77, F	PWD	T2 N1 M1	Total thyroidectomy and 131-I ablation therapy (150 mCi)	L2	Severe pain and neurological symptoms	SET then 131-I therapy (150 mCi)	AWD (40)
	3	63, F	FWD	T2 N0 M1		T7	Severe pain, neurological symptoms and SCC	SET then 131-I therapy (150 mCi)	CR (38)
	4	60, F	FWD	T2 N0 M1		L4	Severe pain, neurological symptoms and SCC	SET then 131-I therapy (150 mCi)	CR (21)
Vitale *et al.* 2001	1	35, F	FWD	-	Not specified; failure of all conventional therapy	Spine	Painful bone metastases	1 year monthly course of I.V. pamidronate (90 mg)	PR (12)
	2	76, F	PWD	-		Spine	Painful bone metastases	1 year monthly course of I.V. pamidronate (90 mg)	AWD (12)
	3	48, F	FWD	-		Spine	Painful bone metastases	1 year monthly course of I.V. pamidronate (90 mg)	PR (12)
	4	46, F	FWD	-		Spine	Painful bone metastases	1 year monthly course of I.V. pamidronate (90 mg)	AWD (12)
	5	54, F	MTC	-		Spine	Painful bone metastases	1 year monthly course of I.V. pamidronate (90 mg)	PD (12)
	6	66, F	MTC	-		Spine	Painful bone metastases	7 monthly course of I.V. pamidronate (90 mg)	PD (12)
	7	61, M	FWD	-		Spine	Painful bone metastases	1 year monthly course of I.V. pamidronate (90 mg)	AWD (12)

DTC; differentiated thyroid carcinoma, PWD; papillary well differentiated, FWD; follicular well differentiated, MTC; medullary thyroid carcinoma, PDTC; poorly differentiated thyroid carcinoma, FLD; follicular less differentiated, UTC; undifferentiated thyroid carcinoma, T; thoracic, L; lumbar, C; cervical, BM; bone metastasis, SM; spinal metastasis, SSC; spinal cord compression, RAI; radioactive iodine (131-I), ERT; external radiotherapy, CT; chemotherapy, SET; selective embolization therapy, TES; total en bloc spondylectomy, ZA; zolendronic acid, NED; no evidence of disease, SD; stable disease, CR; complete remission, PR; partial response, PD; progressive disease, AWD; alive with disease, DOD; death of disease, SRE; skeletal-related events, PFS; progression-free survival, DSS; disease-specific survival, Yrs; years.

**Table 3:** Summary results of studies that deployed conventional therapeutic modalities in patients with distant metastasis from thyroid carcinoma

**Author, Year**	**Number of patients (% female)**	**Median age (yrs) at time of symptomatic distant metastatic disease (range)**	**Thyroid cancer histology (%)**	**Percentage of Patients with bone/spinal metastasis**	**Signs and symptoms of distant metastasis/indication for intervention**	**Intervention for distant metastasis (% of patients)**	**Other metastases (%)**	**Main outcome(s)**
Schlumberger *et al.*, 1999	394 (61.7%)	-	PWD (46.1%), FWD (7.5%), FLD (46.3%)	BM (27.4%), BM + Lung (18.3%)	Pain, swelling, orthopaedic complications	RAI (88%), ERT (35%), surgery (18%), CT (12%)	Lung (54.3%)	5 year/10 year/15 year survival: 55%/40%/33%
Pittas et al. 2000	146 (58%)	58.7	PWD (13%), FWD (12%), Hurthle (6%), UTC (7), PDTC (13%), medullary (4%), lymphoma (2%), unknown (43%)	BM (100%), SM (54%)	Pain (50%), swelling (11.0%), pathologic fracture (4.1%), SCC (3.4%)	RAI (51%), ERT (75%), Surgery (26%), CT (12%)	Other (36%)	5 yr/10 yr survival: 53%/35%
Bernier *et al.*, 2001	109 (71%)	63 (20–87)	PWD (17%), FWD (71%), Unknown (12%)	BM (100%), SM (68%)	Pathological fractures (13%), Radiculalgia (4%), SCC (28%), SCC + fracture (6%r	Complete BM surgery (22%), palliative surgery (55%), ERT (36%), SET (31%), CT (2%)	Lung (34%), thyroid (11.%), other (25%)	5 yr/10 yr/20 yr survival rate: 41%/15%/7%, Mean survival 5.6 years. Remission rate 4%, Mortality 84%
Petrich *et al.*, 2001	107 (28.0%)	62.1	PWD (27.1%), FWD (72.9%)	BM (100%)	-	RAI (100%), ERT (14%)	Lung (41.1%)	CR 25 (23.4%), PR 29 (27.1%), PD 53 (49.5%), Mean survival 7.9 yr
Stojadinovic *et al.*, 2002	260 (51.2%)	58 (5–91)	PWD (58.8%), FWD (34.2%), Hurthle (6.9%)	BM (15%)	-	Surgery (22.7%), + RAI (13.8%), RAI +/− other (54.2%), Other (ERT, CT, supportive) (23.1%)	Lung (32.7%), single site 59 (22.7%), multisite 77 (29.6%)	5 yr DSS: Complete metastasectomy 78%, Partial resection 43%, Non-operative treatment 46%
Zettinig *et al.*, 2002	41 (58.5%)	60 +/− 12	PWD (14.6%), FWD (85.4%)	BM (100%)	Pain (37%), goitre/nodules (51%), dyspnoea (10%)	Surgery (51.2%), RAI (78%), ERT (27%), CT (6%)	-	5 yr/10 yr survival: 69.2%/38.9%
Durante *et al.*, 2006	444 (62%)	-	PWD (42%), FWD (15%), FPD (41%)	BM (26%), BM and lung (18%)	-	RAI + CT, EBR and/or surgery in indicated BM patients	Lung (52%), other (5%)	10 yr survival in negative and abnormal study patients, 92% and 19%, respectively. BM only patients required median cumulative I-131 dose of 250 mCi to attain negative studies
Orita *et al.*, 2010	52 (65.4%)	59 (32–77)	PWD (52%), FWD (48.%)	BM (100%)	Pain (46.2%), mass bone lesions (9.6%), paralysis/numbness (7.7%), fracture (3.8%)	Surgery (21.2%), ERT (75%), RAI (75%), CT (7.7%), Bisphosphonate (34.6%), TSH suppression (36.5%)	Lung (44.2%), pleura (9.6%), brain (5.8%), liver (3.8%)	5 yr/10 yr DSS: 36%/10%
Qiu *et al.*, 2011	106 (62%)	53 (12–85)	PWD (41.5%), FWD (49.1%), Follicular variant of Papillary (9.4%)	BM (45.3%), BM + other (54.7%)	Pain (57.5%), pathological fracture (5.7%), SCC (4.7%)	Oral RAI (100%), Surgery (24.5%)	Cervical lymph nodes (16.0%), lung (42.%), other (20%)	5 yr/10 yr survival rate: 86.5%/57.9%

DTC; differentiated thyroid carcinoma, PWD; papillary well differentiated, FWD; follicular well differentiated, MTC; medullary thyroid carcinoma, PDTC; poorly differentiated thyroid carcinoma, FLD; follicular less differentiated, UTC; undifferentiated thyroid carcinoma, T; thoracic, L; lumbar, C; cervical, BM; bone metastasis, SM; spinal metastasis, SSC; spinal cord compression, RAI; radioactive iodine (131-I), ERT; external radiotherapy, CT; chemotherapy, SET; selective embolization therapy, TES; total en bloc spondylectomy, ZA; zolendronic acid, NED; no evidence of disease, SD; stable disease, CR; complete remission, PR; partial response, PD; progressive disease, AWD; alive with disease, DOD; death of disease, SRE; skeletal-related events, PFS; progression-free survival, DSS; disease-specific survival, yr; year.

**Table 4:** Summary results of studies that deployed novel therapeutic modalities in patients with distant metastases from thyroid carcinomas

**Author, Year**	**Number of patients (% female)**	**Median age (yrs) at time of symptomatic distant metastatic disease (range)**	**Thyroid cancer histology (%)**	**Percentage of patients with bone/spinal metastasis**	**Signs and symptoms of distant metastasis/indication for intervention**	**Intervention for distant metastasis (% of patients)**	**Other metastases (%)**	**Main outcome(s)**
Kloos *et al.*, 2009	Total 56 (44.6%): Treatment group 19 (42%); Control group 37 (45.9%)	-	Papillary (73%), Follicular (4%), Hurthle cell (16%), Anaplastic (7%)	BM (21%)	Evaluate the activity of sorafenib in metastatic thyroid carcinoma	Treatment group; sorafenib 400 mg PO bd	Lung (96), lymph (94%), other (14%)	Partial response in 15% of patients (7.5 months median duration). Median progression-free survival was 15 months
Cabanillas *et al.*, 2010	15 (60%)	61 (38–83)	PWD (53.3%), FWD (33.3%), Hurthle (13.3%)	BM (26.7%)	Evaluate the activity of sorafenib and sunitinib in progressive and radioactive resistant DTC	Sorafenib 200–400 mg PO bd 86.7%, sunitinib 50 mg PO od 13.3%	Lymph (73.3%), lung (66.7%), pleura (13.3%)	SD 9 (60%), PR 3 (20%), PD 3 (20%). 2 yr survival 67%
Orita *et al.*, 2011	Total 50 (72%): Treatment group 22 (68.2%); Control 28 (75%)	59 (32–77)	PWD (52%), FWD (48%)	SM (40%), BM (100%)	Evaluate the efficacy of ZA for treatment of symptomatic BM in DTC	Treatment group; ZA 4 mg IV/month (100%), Surgery (18%), ERT (77%), RAI (50%)	Others (60%)	Treatment group/control SRE-free 3 yr survival: 86%/50%, mortality 54%
Wells *et al.*, 2012	Total 331 (42.6%): Treatment group 231 (42%); Control group 100 (44%)	-	Advanced MTC (100%)	Treatment group BM (34%), control group BM (40%)	Evaluate the efficacy of vandetanib in advanced or metastatic MTC	Treatment group; vandetanib 300 mg PO	Hepatic (46.5%), lymph nodes (40.8%), respiratory (38.1%), neck (10.0%)	Significant prolongation of PFS with vandetanib compared with placebo. PD 37%, mortality 15%

DTC; differentiated thyroid carcinoma, PWD; papillary well differentiated, FWD; follicular well differentiated, MTC; medullary thyroid carcinoma, PDTC; poorly differentiated thyroid carcinoma, FLD; follicular less differentiated, UTC; undifferentiated thyroid carcinoma, T; thoracic, L; lumbar, C; cervical, BM; bone metastasis, SM; spinal metastasis, SSC; spinal cord compression, RAI; radioactive iodine (131-I), ERT; external radiotherapy, CT; chemotherapy, SET; selective embolization therapy, TES; total en bloc spondylectomy, ZA; zolendronic acid, NED; no evidence of disease, SD; stable disease, CR; complete remission, PR; partial response, PD; progressive disease, AWD; alive with disease, DOD; death of disease, SRE; skeletal-related events, PFS; progression-free survival, DSS; disease-specific survival, yr; year.

One of our studies, by Wells *et al.*[46], was a randomized controlled trial, which investigated the effect of vandetanib versus placebo, however, there were no interventional trials comparing the effect of different treatments of metastatic disease. Of the retrospective trials, too, no studies sought to compare treatments.

Two studies looking at the specific use of bisphosphonates were Orita *et al.*[15] and Vitale *et al.*[45], although Orita *et al.* had mentioned, previously, that bisphosphonates contribute to an improved 5-year survival (52% with and 30% without bisphosphonate; *P* value = 0.11). However, on subsequent interrogation it was found that zolendronic acid infusion once a month, increased the chance of avoiding skeletal-related events (SREs), defined as bone fracture, spinal cord compression, and hypercalcaemia, in the first 3 years post detection (50% without bisphosphonate compared to 86% with bisphosphonate; *P* value of 0.002). This corroborated Vitale *et al.*’s work that illustrated pamidronate IV infusion once a month, for 12 months, improves the quality of life using the FACT-G scoring system (*P* value = 0.0059) [45].

Surgical intervention for bone metastasis was examined in 4 studies: Bernier *et al.*[34], Demura *et al.*[13], Orita *et al.*[38] and Quan *et al.*[40]. Bernier *et al.*, showed through multivariate analyses that complete SM surgery was an independent prognostic indicator of improved survival, however, this only applied to younger patients (under 45 years of age) [34]. Demura *et al.*, who employed an en-bloc resection approach, reported that the survival rate from surgery was 74% at 5 years and 25% at 10 years. The risk of local recurrence after debulking surgery was 57% and required further surgery at an average of 41 months, however recurrence after total en bloc spondylectomy (TES) was 10% and this difference was statistically significant. They reported that all patients alive at study close were neurologically preserved [13]. Orita *et al.* reported 5-year survival with surgery of 60% compared to 37% (*P* value = 0.05) with no surgery [38].

Selective Embolisation therapy (SET) therapy was investigated in 3 studies, by Van Tol *et al.*[44], Eustatia Rutten *et al.*[16] and, most recently, by De Vries *et al.*[36]. Van Tol *et al.*[15] found that those who received combined embolisation with 131-I therapy, serum Tg fell by 88.7%, which was significantly more than the control group 16.6%; p < 0.05. Eustatia-Rutten *et al.* found it was effective at palliation in 59% of cases, with success duration being 6.5 months alone, but for embolisations combined with additional radioiodine or external irradiation, this was increased to 15 months (P > 0.0146) [16]. Finally, De Vries *et al.*, came to the conclusion that survival is not significantly improved, however symptoms were reduced in 55% of procedures [36].

The impact of ^131^I therapy was investigated in 5 studies [14,29,38,39,41]. Although Pittas *et al.*[29] did not find an increased survival in those who were treated with ^131^I for bony metastasis, it was suggested that it may be beneficial in patients whose bony metastases show ^131^I uptake. Petrich *et al.*[39] showed positive radioiodine uptake by metastases significantly increased survival compared to those individuals who had negative uptake. Orita *et al.*[38] and Hindie *et al.*[14] provided findings that a significant survival advantage was obtained with ^131^I therapy supporting that claim. Additionally, a study by Qiu *et al.*[41] found that ^131^I therapy was 63.9% effective at reducing symptoms due to bony metastasis such as pain, and could therefore be used as a palliative agent in patients with multiple bone metastases.

VEGFR therapy was investigated in two studies [37,46] in the form of sorafenib and vandetanib. Kloos *et al.*[37] found that sorafenib caused stabilisation of disease progression for greater than 6 months in 56% of patients (95% CI, 40 to 72). Wells *et al.*[46] investigated vandetanib in the form of a phase III randomised control trial and investigated the progression free survival (PFS) in comparison with placebo. They found a prolonged progression free survival in the treatment group estimated at 30.5 months, compared a PFS of 19.3 months in the placebo group. However, 12% of patients stopped the drug due to adverse events [46].

## Discussion

Metastatic thyroid tumours cause considerable morbidity [48]. They are also indicative of advanced disease and are associated with a poor prognosis [29] and a reduction in treatment response. However, comparatively, they have a more favourable prognosis of most tumours, which metastasize to the spine [40,49]. Tumour invasion of the vertebral body compromises the supportive structure of the spine [40]. Compression of the cord or cauda equina induces severe pain, paralysis and sphincter dysfunction [40,48]. Therapeutic interventions should, therefore, target first and foremost the integrity of the spine to prevent neurological complications. In doing so, they may vouchsafe symptomatic control, such as pain, paralysis and the impact these have on activities of daily living [48].

Once spinal metastasis has been diagnosed, there are a several modalities of treatment. Treatment can be palliative or curative. The use of radioiodine ablation therapy, selective embolisation therapy (SET), bisphosphonates, surgery, and small molecular therapy is being discussed [40].

Approximately 90% of patients with spinal metastasis experience pain [25]. The pain can be significantly debilitating and interfere with quality of life. Pain, in metastatic cancer, is managed by a multidisciplinary team; treatments include analgesia, bisphosphonates, radiotherapy and chemotherapy [50]. In terms of spinal metastasis, intractable pain is often a surgical indication [13], and many other treatments such as SET and radioiodine have been shown to reduce pain scores [44].

### Radioiodine ablation therapy

Radioiodine therapy is a mainstay of thyroid cancer management [10,19,31]. Many studies reported benefit retrospectively. Intriguingly, Van Tol *et al.*[44] demonstrated that radioiodine ablation reduces the pain rating on a 4 point analogue scale, irrespective of whether SET was combined, in a number of patients with spinal metastasis. Radioiodine absorption is a prognostic factor in metastatic disease [14]. Tumours, which are non-functioning, represent an entity further down the pathway of malignant transformation and they are resistant to the radiation dose delivered through radioiodine sequestration. Hindie *et al.* recommend that young patients who have ^131^I avid metastasis, of a papillary or follicular, well differentiated nature, should receive ^131^I. Durante *et al.*[30] observed that once a patient achieved a positive scan, it on average took a cumulative dose of up to 600 mCi to achieve a negative scan. It has been shown that a dose exceeding this value can lead to increased risk of haematological malignancy, and salivary gland dysfunction. They suggested, therefore, that if patients have been treated with 600 mCi, as a cumulative dose, deciding to re-treat should be made on an individual basis.

### Selective embolisation therapy

SET represents an attractive therapy due to its induction of rapid, albeit, transient symptom amelioration. Eustatia Rutten *et al.*[16] suggested that symptomatic control and progression arrest is achievable in 59% of embolizations. In reality, however, this does not equate to 59% of patients receiving neurological and pain benefit. They also found that the benefit lasted a mean duration of 6.5 months suggesting that more sessions may be required to continue to ameliorate symptoms [16]. There is evidence, albeit sparse, of its efficacy in cervical instability [16,36]. Serum thyroglobulin (Tg) a marker of tumour burden, was radically reduced in an a study by Van Tol *et al.* in the embolisation group, (88.7% c.f. 18.6% in the control; p < 0.05). Of course, this is only a proxy for disease. De Vries *et al.*[36] postulated that embolisation may be beneficial in not only palliating symptoms, but can be used in an adjuvant setting, a form of pre-operative embolisation to help reduce blood loss^37^. Unfortunately, SET does not appear to improve survival [16,36]. SET alone induces tissue hypoxia, a stimulus for tumour growth. The combination of SET with radiation therapy may confer a synergistic benefit.

### Surgery

The role of surgery is debatable. Quan *et al.*[40] suggests that surgery is indicated for patients with intractable pain, cord compression, neurological deficit or cervical instability [40]. Cervical metastasis can produce pain unresponsive to medical management, which can affect activities of daily living. In addition neurological deficits and pathological fractures can occur due to metastatic lesions reducing the integrity of the vertebral bodies [50]. Metastatic cervical spine disease causing instability is usually treated with anterior reconstruction and stabilisation [51] Bernier *et al.*[34] reported that surgery as a whole did not improve survival, but complete bone metastasis (BM) resection did [34]. A factor for deciding which type of surgery patients were given was age. Surgical treatment in the young should, therefore, be more aggressive. Demura *et al.*[13] suggested that total en bloc spondylectomy (TES) may provide better local control of thyroid cancer spinal metastasis, compared with debulking surgery. Complete resection of synchronous metastasis was associated with increased survival [13]. The authors showed a significant difference in the risk of local recurrence with debulking surgery (risk of recurrence 57%, c.f. 10% with TES). Patients who underwent complete metastasectomy had significantly improved survival than those having palliative resection (5-year DSS, 70% vs. 30%, *P value* = 0.004) [43]. This indicates, perhaps, that it is important to treat as many metastasis burden as possible, however, this may not always be achievable. Although, there is considerable bias in this conclusion, in that those patients whose disease is too extensive to treatment would, of course, have a poorer survival. Kushchayev *et al.*[23] also mentioned percutaneous vertebroplasty for patients who cannot be operated on but at risk of fractures.

### Bisphosphonates

Bisphosphonates have shown some clinical usefulness in metastatic bone disease in terms of symptom control. Orita *et al.*[15] illustrated that skeletal related events occurred in significantly lower frequency in patients on zoledronic acid (3/22 patients, 14%) than without (14/28 patients, 50%; p > 0.007). Pamidronate was also effective at pain amelioration [45], with a 31.35% reduction using the VAS scoring. Patient tolerability was good, although the side effect of osteonecrosis of the jaw is, unfortunately, relatively high at 9% [15], though the study size was small. Other studies have intimated that as much as 5% of the oncological patients receiving bisphosphonate therapy for ~4.4 years develop bisphosphonate-related osteonecrosis of the jaw (BRONJ) [52]. The authors reported an impressive spinal cord compression reduction, although study size was small (n = 28).

### Small molecules

The effect of small molecule inhibitors has revolutionised the treatment of some cancers, and the search of molecular targets in neoplasm continues. Growth signaling pathways are implicated in tumorigenesis, especially those of angiogenesis. Vascular endothelial growth factor receptors (VEGFR) inhibitors appear not to improve progression free survival in metastatic thyroid cancer [37]. However, small study sizes have impeded research. In a study by Kloos *et al.*[37], side effects prompted 52% of patients to reduce their dose. Cabanillas *et al.*[35], treated 15 patients, mostly with non ^131^I avid disease with different VEGFR inhibitors, notably sorafenib. Interestingly, progression free survival was increased. It was observed that patients whom had their SM irradiated, prior to VEGF therapy, maintained stable bone disease, whereas those who did not have irradiation experienced rapid progression, despite a good response of their lung metastasis to VEGF therapy. This raises the possibility that treating patients with (external bean radiation therapy) EBRT prior to VEGF therapy could improve survival. This may need to be examined in further detail.

### Difficulties encountered

We encountered difficulties with regards to study selection and data extraction, contemporaneous research, study size and consistency of protocol. We cannot claim to have used a complete and exhaustive list of studies. Our search results yielded a large body of research relevant to our title. We cross-examined the references to add to this body. This approach is rigorous but not consummate.

In addition, many of the papers made analysis difficult due to the presentation of their data. Spinal metastasis was rarely isolated as a subgroup. Indeed, many forms of metastases were pooled for the statistics. It was, therefore, impossible to extract specific and valuable information from these studies [35,37,46].

Several retrospective studies did not use contemporary data sets. Studies, recruited by the inclusion criteria, analysed investigation and treatment modalities in a patient pool, which was considerably dated due to some cohorts having been accrued over decades. These studies spanned a long period of time, during which, treatment and investigative modalities had significantly evolved. They, therefore, encompassed a number of out-dated clinical practices and ignored currently well-established modalities [29,30,34,42].

Much of the literature on spinal metastasis in thyroid cancer is based on small sample sized studies. This makes it difficult to measure the effectiveness of some treatment modalities and draw conclusions. Additionally, this makes it difficult to use the data in multivariate analysis due to the high standard errors, which would occur with small sample size [14,16,36,40,44,45].

Finally, most countries have specific guidelines and recommendations for the management of certain cancers, including the UK [19]. It was difficult to make recommendations when papers preconised the use of different ‘successful’ methods in similar situations. This applied to certain treatments, such as thyroidectomy, or to dose, such of ^131^I used [14,16,19,40].

### Recommendations

**Figure 2:**
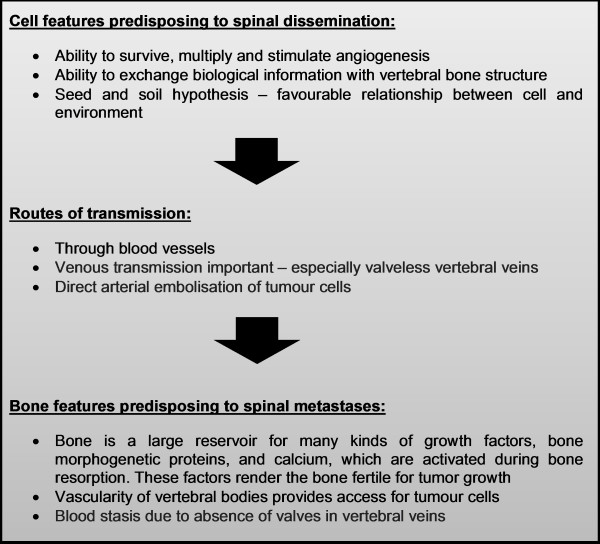
Route of transmission from thyroid to spine.

**Figure 3:**
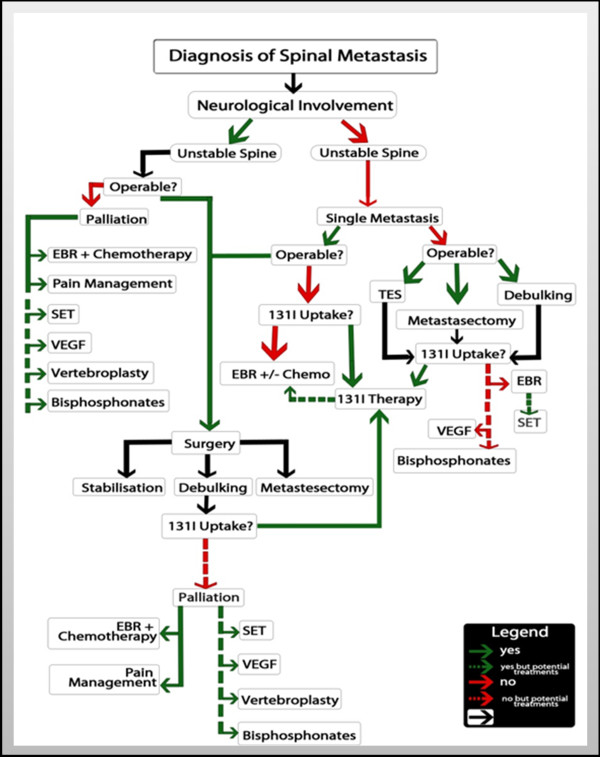
The proposed recommendations of treatment.

Individual prognosis depends upon age at diagnosis of spinal metastasis, tumour burden and number of spinal metastases [29,30,34,42,47]. Early detection is a prognostic factor in spinal metastasis of differentiated thyroid carcinoma (DTC). This early detection could help prevent dissemination to the spine. The proposed process of which is outlined in Figure 2. Post-surgical ^131^I ablation, in addition to adjuvant therapy, has been shown to aid in the early detection of bone metastasis. At this time, thyroglobulin levels are low, and radiological studies are often negative [14]. Treatment of metastasis should be done in a multi-disciplinary fashion and take into account tumour response, palliation and neurological function. Recommendations of treatment are illustrated in Figure 3. 

Radioiodine absorption is a prognostic factor in metastatic disease. Multivariate analysis expounds the prognostic benefit of complete metastectomy and ^131^I-therapy, when applicable. Survival rates in DTC patients are improved by these interventions [13,34,38,47]. This is, especially so in those below the age of 45, in whom bone is the only distant metastasis [34]. We espouse that therapy should be aggressive, particularly in the young [34].

Patients below the age of 45 years of age with small DTC distant metastasis should be treated with radioiodine until there is an uptake decline or a cumulative dose of 600 mCi (22 GBq) reached [30]. Subsequent courses of radioiodine treatment, at least one year after initial radioiodine treatment may also be a recommended.

Radioiodine therapy comes with its limitations, however. Treatment exceeding 600 mCi may be deleterious to the health. There is a significant increase in salivary gland dysfunction, cancer and leukaemia in patients. In patients with continued uptake, a decision to continue treatment beyond that dose should be considered on a case-by-case basis, due the risks associated [30].

In patients with non ^131^I avid disease, or who are ^131^I non-responders, other treatment modalities ought to be evaluated. Such treatments include combination therapy, SET or small molecules, along with radiotherapy. These may assuage symptomatic disease. VEGFR is particularly helpful in ^131^I non-avid disease. SET and bisphosphonates are especially useful modalities in reducing bone pain [15,16,44,45].

Thyroid cancer pathogenesis and promulgation to the bone has yet to be fully elucidated despite some novel research by Torre *et al.*[53] into the role of angiogenic and lymphangiogenic phenotypes, spread to the vertebral column is poorly understood. Dissection of this journey opens the possibility of intervening to improve patient survival and reduced morbidity.

## Conclusions

There is a need for more histology-specific research in the field of thyroid carcinoma metastasis. While study sizes are difficult to control, we believe that there is room for improvement in study design and in the quantity of research on specific treatment modalities.

To sum up, for a young patient aggressive surgery is the best first line management. Radioiodine and surgery is our best combined management. Combination therapy, SET or small molecular inhibitor with radiation is recommended.

Furthermore, VEGFR is particularly helpful in ^131^I non-avid disease. SET and bisphosphonates are useful modalities in palliation. However, more research is required into the effect of combination therapies.

Future prospects look towards intersecting the molecular pathways of tumourigenesis and dissemination. Indeed, spread to the spine is haematogenous, which opens an avenue to pursue. At present, surgery is our most logical solution, but it certainly is not curative, but, rather, symptom control. Cure is the aim.

## Competing interests

The authors declare that they have no competing interests.

## Authors’ contribution

All authors contributed to conception and design, carried out the literature research, manuscript preparation and manuscript review. All authors read and approved the final manuscript.
